# The Association Between Telehealth Utilization and Policy Responses on COVID-19 in Japan: Interrupted Time-Series Analysis

**DOI:** 10.2196/39181

**Published:** 2022-07-12

**Authors:** Tomoki Ishikawa, Jumpei Sato, Junko Hattori, Kazuo Goda, Masaru Kitsuregawa, Naohiro Mitsutake

**Affiliations:** 1 Institute for Health Economics and Policy Minato-ku, Tokyo Japan; 2 Faculty of Health Sciences Hokkaido University Sapporo, Hokkaido Japan; 3 Institute of Industrial Science The University of Tokyo Meguro, Tokyo Japan

**Keywords:** telehealth, COVID-19, health services research, interrupted time series

## Abstract

**Background:**

Telehealth using telephones or online communication is being promoted as a policy initiative in several countries. However, there is a lack of research on telehealth utilization in a country such as Japan that offers free access to medical care and regulates telehealth provision—particularly with respect to COVID-19.

**Objective:**

The present study aimed to clarify telehealth utilization, the characteristics of patients and medical institutions using telehealth, and the changes to telehealth in Japan in order to support the formulation of policy strategies for telehealth provision.

**Methods:**

Using a medical administrative claim database of the National Health Insurance and Advanced Elderly Medical Service System in Mie Prefecture, we investigated patients who used telehealth from January 2017 to September 2021. We examined telehealth utilization with respect to both patients and medical institutions, and we determined their characteristics. Using April 2020 as the reference time point for COVID-19, we conducted an interrupted time-series analysis (ITSA) to assess changes in the monthly proportion of telehealth users to beneficiaries.

**Results:**

The number of telehealth users before the reference time point was 13,618, and after the reference time point, it was 28,853. Several diseases and conditions were associated with an increase in telehealth utilization. Telehealth consultations were mostly conducted by telephone and for prescriptions. The ITSA results showed a sharp increase in the proportion of telehealth use to beneficiaries after the reference time point (rate ratio 2.97; 95% CI 2.14-2.31). However, no apparent change in the trend of increasing or decreasing telehealth use was evident after the reference time point (rate ratio 1.00; 95% CI 1.00-1.01).

**Conclusions:**

We observed a sharp increase in telehealth utilization after April 2020, but no change in the trend of telehealth use was evident. We identified changes in the characteristics of patients and providers using telehealth.

## Introduction

A patient’s access to health care services is a major factor affecting health care outcomes. Telehealth is the provision of health care services to patients from providers at different locations using telephone or video consultations. By improving patients’ geographic access or saving on travel costs and time, telehealth reportedly enhances efficacy in increasing continuity of care and medication adherence [1,2]. With its strong contribution to follow-up, telehealth can be widely applied from acute diseases to chronic conditions [3]. Thanks to its administrative benefits, the merits of adopting telehealth have been reported from the viewpoints of both patients and providers: reducing the use of resources in health care facilities, improving access to care, and—notably—minimizing the risk of direct infection transmission [4].

The advantages of telehealth have been reported. However, its effectiveness and availability in real-world settings need verification: External environmental changes and stakeholder decisions (eg, adaption of medical institutions and patient behavior) can affect the utilization of telehealth services [5]. As a key discussion related to effectiveness, some countries (or payers) have limited the coverage of telehealth services to patients living in rural areas [6]. From the standpoint of considering these differences in delivery systems by insurer, additional research would be needed to evaluate how evidence on telemedicine applies in a health care delivery system to which patients have free access, as in Japan. As a preliminary step, an assessment of the prevalence of telehealth is important information not only to support in test evidence but also to evaluate its relevance to policy.

The Japanese government's conservative strategy for telehealth, in conjunction with its various policy responses to COVID-19, have faced a watershed in the years after 2020. With some exceptions, telehealth in Japan has been applied only as a complement to routine care for the sake of ensuring diagnostic accuracy [7]. Similarly, over the past 2 decades, using telehealth for initial consultations has been limited in Japan. In recent years, the Japanese government has promoted telehealth as part of a national growth strategy for information and communication technology; however, regulations have prevented its widespread use [8]. Since early 2020, COVID-19 and associated policy responses have promoted the wider application of telehealth. Many countries and insurers have expanded telehealth services to facilitate patient access to medical services or prescriptions under partial quarantine (eg, school closures and government-imposed social distancing restrictions) [9-12]. Similarly in Japan, regulations on the application of telehealth have been relaxed amid measures such as declaring a state of emergency, closing schools, and triaging for outpatient visits. To increase the number of medical institutions providing telehealth and maintain the delivery of medical care to self-isolating patients, Japan temporarily lifted restrictions on telehealth for initial consultations [13]. The Japanese government has developed these measures to promote telemedicine as temporal measures but made them permanent starting in April 2022. However, the system has been designed to reimburse medical institutions more for face-to-face consultations than for telehealth, which raises concerns about their widespread use. Thus, it is necessary to examine how those measures enacted during the pandemic have affected health care provision in Japan.

Despite the importance of discussions about the future provision of telehealth, there is a lack of data on telehealth use in Japan since COVID-19. In the present study, using the Japanese health care claims database, we examined the following: (1) whether telehealth utilization has increased since COVID-19 and the associated institutional response, (2) whether trends in telehealth use after COVID-19 have increased or decreased, and (3) whether characteristics of telehealth users and providers have changed before and since COVID-19. In order to support the formulation of policy strategies for telehealth provision, we also discuss the future of telehealth provision in Japan, which is regarded as a highly accessible environment for health care with no restrictions on patient access to visit medical facilities of their own choosing.

## Methods

### Data Source

In this retrospective cohort study, we analyzed data from the administrative database of health insurance claims in Mie Prefecture, Japan. Health insurance is mandatory for residents in Japan. The database stores information related to the National Health Insurance and Advanced Elderly Medical Service System; those covered are self-employed individuals as well as retirees and their dependents aged 75 years or above. These insured under that scheme amounted to 37.8% of the total Japanese population in 2020 [14]. The claims data include diagnoses, use of medical care (face-to-face or telehealth), prescriptions, and expenditures related to medical procedures.

The diagnosis information is recorded using the International Classification of Diseases 10th Revision (ICD-10) codes and Japanese diagnosis codes, with flags indicating the diagnosis. With links to insurance information, the database includes patients’ sex, date of birth, and place of residence.

**Figure 1:**
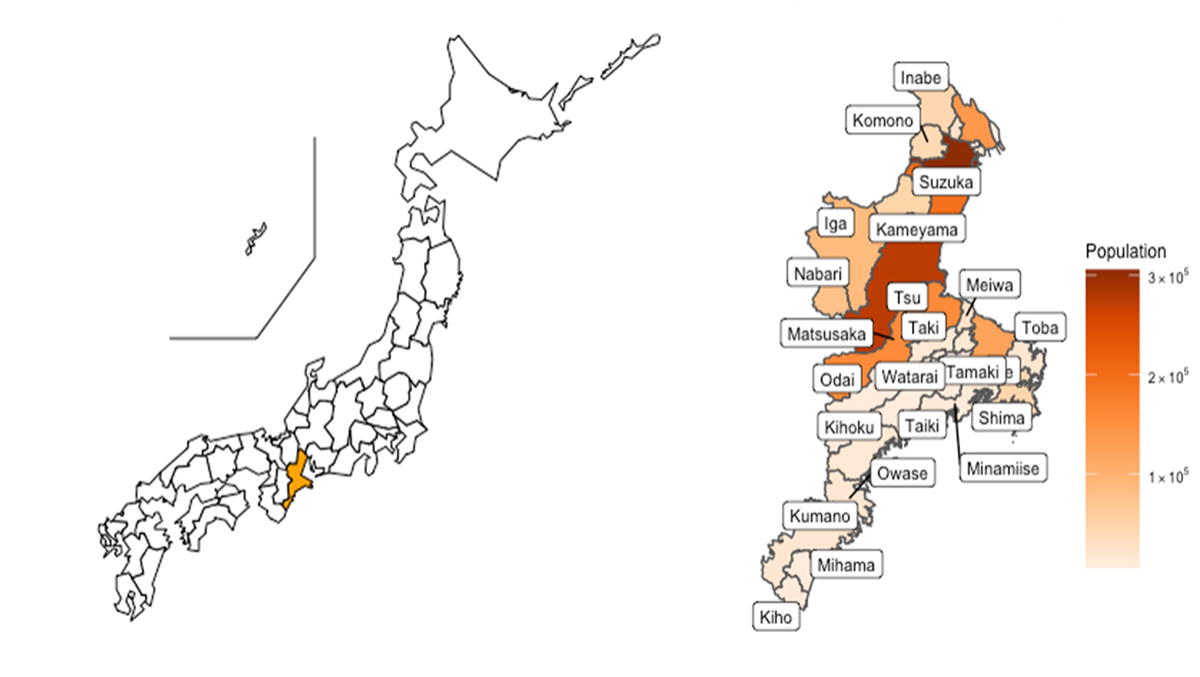
Geographic and demographic details of Mie Prefecture in 2020.

We conducted this study in Mie Prefecture. The prefecture is located toward the center of Japan; it had a population of 1.77 million (22nd highest among the 47 prefectures) and an aging rate of 29.4% (18th highest) [15]. Figure 1 shows the geographic and demographic details for Mie Prefecture in 2020; Table 1 presents information about the medical facilities.

**Table 1:** Comparison of the population and medical facilities between Mie Prefecture and all of Japan in 2020.

Item	Mie Prefecture	Japan
Population, n	1,770,254	126,146,099
Population aged 65 years and over, n	522,073	35,335,805
Aging rate, %	29.4	28.4
Clinics per 100,000 population, n	45.81	44.81
Hospitals per 100,000 population, n	5.31	6.49
Physicians per 100,000 population, n	225.45	250.83

### Participants

The term “telehealth” is polysemous and defined differently in different contexts. In this study, we categorized telehealth services as follows: online (using videoconferencing systems or devices), telephone, and monitoring (using a monitoring device such as a cardiac pacemaker and continuous positive airway pressure). We identified individuals who used any telehealth service at least once from January 2017 to September 2021. We determined the role of telehealth for each patient, such as for prescriptions and home care for COVID-19. Furthermore, we obtained data about sex, age when the patient used the service, and diagnoses. To calculate the monthly proportion of people using telehealth services, we identified the number of patients who received face-to-face consultations (visits to clinics or hospitals). The diagnoses were categorized by diseases according to ICD-10. We followed previous studies regarding the selection of diseases of interest for telehealth, mapping the targeted diseases, and ICD-10 classification [16,17]. We summarized the following: sex, age, type and frequency of telehealth services used, medical expenses recorded with the same claims as used with telehealth, and disease or condition when patients used telehealth. We determined the characteristics of medical institutions that provided telehealth at least once with respect to clinics or hospitals, number of beds, and location (within or outside Mie Prefecture) using a unique number code applied to medical institutions.

We compared the characteristics of telehealth users for a year before and after April 2020 as the reference time point, which was the first declaration of the state of emergency in Japan [18]. We also identified patients who used telehealth for initial consultations following the Japanese government announcement on April 10, 2020, that patients could do so.

### Ethics Approval

The data set included in the study was provided by the regional public health care insurers in Mie prefecture with the approval of the personal information protection commission in charge of each of the local government areas and by contract with the local governments and the insurers.

### Outcome

The outcome was a change in the monthly proportion of telehealth users to beneficiaries. We evaluated the outcomes by stratifying patients into 2 age groups: 65 years old and older and younger than 65 years. To evaluate the impact of each disease, we applied stratification based on diseases and conditions. The secondary outcome was the change in the monthly proportion of telehealth users to beneficiaries.

### Statistical Analysis

We compared continuous variables for the 2 groups using the Wilcoxon rank sum test. Categorical data (such as sex) were compared using the chi-square test with Yates correction.

To determine the outcomes, we undertook an interrupted time-series analysis (ITSA) [19,20] to evaluate the association between the reference time point and the outcomes from January 2017 to April 2021. Also referred to as segmented time-series regression, ITSA is applied to statistically measure changes in various levels and trends in a postintervention period compared with a pre-intervention period [21]. Owing to the frequency of telehealth use, we used ITSA in the present study with a log-linear model as the link function and assumed a Poisson error structure with adjusted seasonality, which could also be called a segmented time-series Poisson regression:




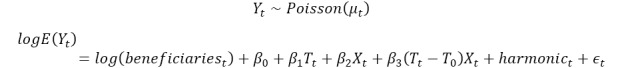




*Y_t_* represents the monthly number of telehealth users to beneficiaries at time point *t*; *beneficiaries_t_* signifies the number of monthly beneficiaries. We set it to calculate the proportion of telehealth users to beneficiaries as a log-offset term. *T_t_* represents the time since January 2017; *T_0_* is a dummy variable signifying *t* at the reference time point, the 40th month in this study. *X_t_* is a dummy variable indicating the time after the reference time point: After April 1, 2020, it was 1; before, it was 0. *β*_0_ signifies the level of *Y_t_* at *t*=0. *β*_1_ represents the pretrend; *β*_2_ is the outcome change immediately after the outbreak of COVID-19 in Japan and the policy responses to it; *β*_3_ is the change in the trend compared with before the reference time point. *harmonic*_t_ was set as the Fourier term to adjust for seasonality [19].

To remove the effect of an immediate increase at the reference point, we excluded 1 month’s observations before and after that point [22]. For subgroup analysis, the same Poisson regression was applied to the groups under 65 years old and 65 years old and older. For the sensitivity analysis, we changed the bandwidth for the removal for the overall telehealth user population from 0 months to 2 months; we did so to assess robustness and goodness of fit for each model based on the Akaike information criterion (AIC).

We performed all statistical analyses using R version 4.1.0 (R Foundation for Statistical Computing, Vienna, Austria). All statistical tests were 2-sided; we set the significance level at 5%, and we used 95% CIs for interval estimations of the outcomes.

## Results

The characteristics of patients who used telehealth services appear in Table 2. The number of users before the reference time point was 13,618; after that time point, the number increased to 28,853 (111.87% growth). At any time, there were more female patients. We observed increases in the following for using telehealth services: frequency of annual use per patient (average from 1.41 to 2.26), proportion of users for initial consultation (from 0.2% to 2.2%), and medical expenses (median from 12,550 yen to 15,020 yen).

Regarding the mean values for telehealth, over 90% (12,905/13,618, 94.8% before April 2020; 26,331/28,853, 91.3% after April 2020) of patients received a consultation by telephone. There was an increase in the use of monitoring equipment after the reference time point (from 707/13,618, 2.5% to 2511/28,853, 8.8%). Online consultation was rarely used: Only 0.8% (11/28,853) of the sample used it after the reference time point. For over 90% (12,943/13,618, 95.0% before April 2020; 26,494/28,853, 91.8% after April 2020) of cases, telehealth was used for prescriptions.

The characteristics of medical facilities where telehealth services provided appear in Table 3. The number of medical facilities that provided telehealth before the reference time point was 1007; after that time point, the number increased to 1392 (38.2% growth). Regarding the type of facility, the number of hospitals increased; regarding location, the ratio of services provided to patients within the same prefecture to those provided to out-of-prefecture patients reversed, and the number of services provided to out-of-prefecture patients increased.

**Figure 2:**
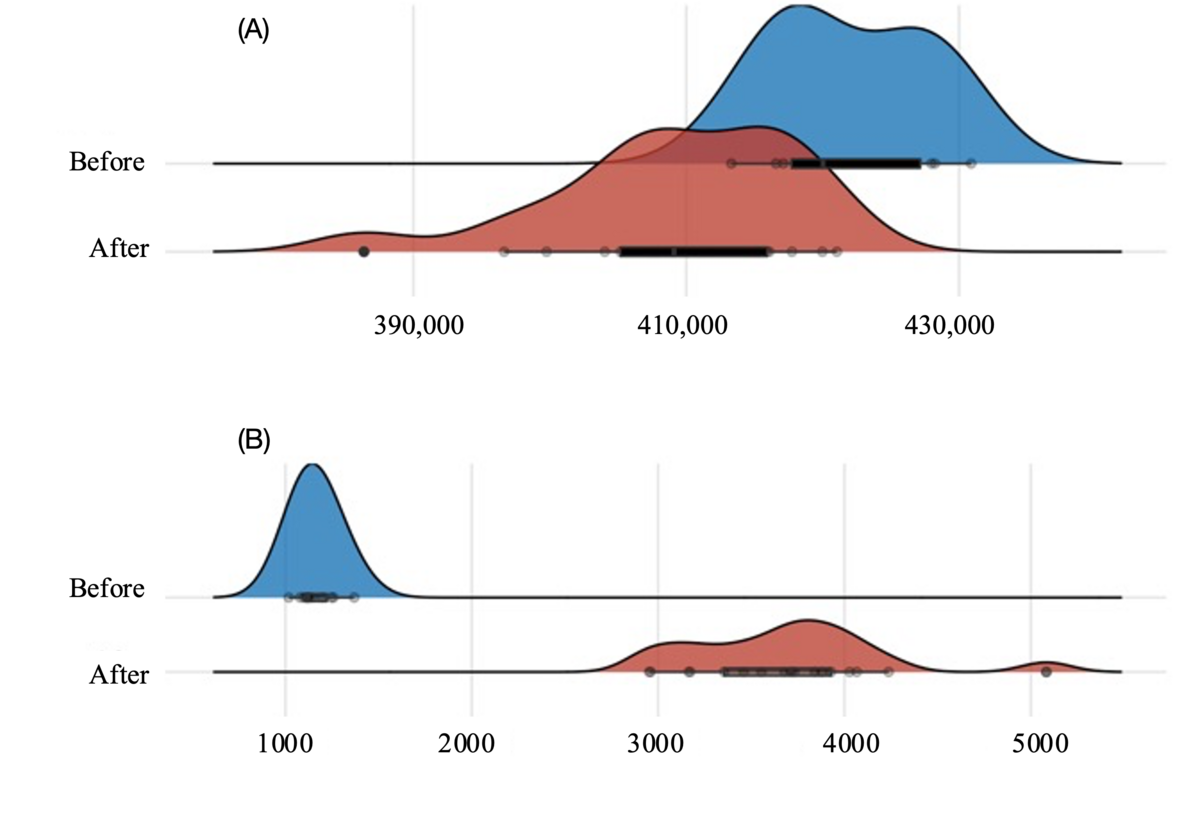
Density plot and boxplot of monthly numbers of (A) telehealth and (B) face-to-face consultations before and after April 2020.

Figure 2 presents the density distribution of the frequency of monthly telehealth use and face-to-face visits. Telehealth use showed an increase after the time reference point compared with before; face-to-face care evidenced a decrease.

**Figure 3:**
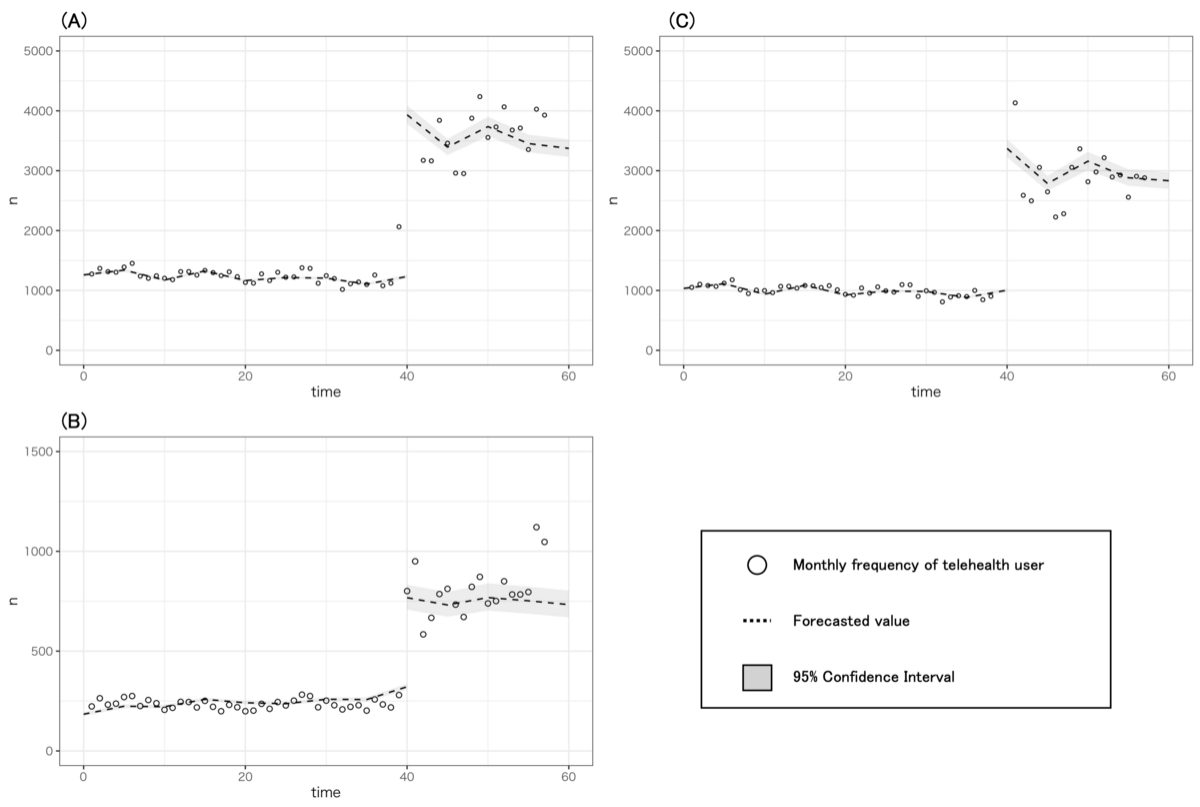
Monthly frequency of telehealth used (scatter plot) by (A) everyone, (B) those aged at least 65 years, and (C) those aged less than 65 years, with Poisson regression adjusted seasonality (dashed lines).

Figure 3 presents the monthly frequency of telehealth use during the study period. The dashed lines in that figure indicate the ITSA estimation results of a Poisson regression model, adjusted trend as seasonality. We observed an immediate increase in the proportion of telehealth users to beneficiaries after the Japanese government first declared a state of emergency for COVID-19.

We evaluated the immediate increase after the reference time point and the change in the trend of telehealth use using the rate ratio (RR) through the ITSA (Table 4). The RR for overall telehealth use during COVID-19 (April 2020 to September 2021) compared with pre-COVID-19 (January 2017 to March 2020) was 3.23 (95% CI 3.14-3.31). Thereafter, the frequency of telehealth use during COVID-19 was substantially unchanged (change in trend 1.00; 95% CI 1.00-1.01). In the subgroup of users aged over 65 years, the RR for the immediate change was 3.25 (95% CI 3.15-3.34), and the change in the trend was 1.00 (95% CI 1.00-1.00). Similarly, in the subgroup of users aged under 65 years, the immediate change was 2.48 (95% CI 2.35-2.62), and the change in the trend was 1.00 (95% CI 1.00 to 1.01). As shown in Multimedia Appendix 1, no autocorrelation and association with the number of COVID-19 confirmed patients was found for the number of patients who used telemedicine over time.

The results of our sensitivity analysis appear in Table 5. In all cases, the RR and its CI consistently exceeded 1. Without removal adjustment, the AIC was highest at 8390.9; the AIC was lowest in models with the removal bandwidth at 1 month before and after the time reference point (base model).

**Table 2:** Characteristics of patients who used telehealth at least once for consultations before and after April 2020.

Characteristics		Before April 2020	After April 2020	*P* value
Unique patient IDs with confirmed telehealth use, n		13,618	28,853	–^a^
Age (years), mean (SD)		73.38 (17.58)	72.26 (18.30)	<.001
Female, n (%)		8569 (62.9)	17,629 (61.1)	<.001
Frequency of telehealth use per user, mean (SD)		1.41 (1.12)	2.26 (2.49)	<.001
Users who used telehealth for initial consult, n (%)		76 (0.2)	622 (2.2)	<.001
Medical expense at the same claims (yen^b^), median (IQR)		12,550 (4450-27,842.5)	15,020 (5940-35,720)	<.001
**Type and role of telehealth services, n (%)**				
	Online	6 (0.4)	11 (0.8)	.29
	Monitoring	707 (2.5)	2511 (8.8)	<.001
	Telephone	12,905 (94.8)	26,331 (91.3)	<.001
	Prescription	12,943 (95.0)	26,494 (91.8)	<.001
	Home care for COVID-19^c^	–	213 (0.7)	–
**Disease or condition, n (%)**				
	Anemia	298 (1.8)	502 (1.7)	.20
	Arthritis	264 (1.6)	438 (1.5)	.16
	Cancer	69 (0.4)	147 (0.5)	.38
	Cerebrovascular disease	133 (0.8)	207 (0.7)	.13
	Asthma	193 (1.2)	274 (0.9)	.005
	Chronic kidney disease	49 (0.3)	98 (0.3)	.73
	Coronary heart disease	237 (1.5)	487 (1.6)	.21
	Dementia	127 (0.8)	323 (1.1)	.003
	Diabetes	386 (2.4)	720 (2.4)	.96
	Hyperlipidemia	412 (2.6)	940 (3.1)	<.001
	Cardiovascular disease	1471 (9.1)	3162 (10.6)	<.001
	Hypertension	377 (2.3)	781 (2.6)	.08
	Hyperuricemia	126 (0.8)	252 (0.8)	.52
	Spine disorder	533 (3.3)	810 (2.7)	<.001
	Digestive system disorder	1791 (11.1)	3488 (11.7)	.08
	Mental disease	277 (1.7)	1031 (3.4)	<.001

^a^Not applicable.

^b^US $1 = 118.48 yen on April 1, 2020.

^c^Number of claims based on incentives granted by the Japanese government for telehealth to COVID-19 patients receiving home or overnight care (August 16, 2021); includes both initial consultations and follow-up.

**Table 3:** Characteristics of medical facilities where telehealth services were used at least once before and after April 2020.

Characteristics			Before April 2020		After April 2020		*P* value
Medical institutions with unique IDs confirmed to have provided telehealth, n			1007		1392		–^a^
**Type of facility^b^, n (%)**							
	Clinic	891 (88.5)		1173 (84.3)		.004	
	Hospital	116 (11.5)		219 (15.7)			
**Number of beds, n (%)**							
	<20	891 (88.5)		1173 (84.3)		.10	
	20-100	36 (17.9)		61 (19.1)			
	100-200	33 (16.4)		67 (21.0)			
	200-300	17 (8.5)		33 (10.3)			
	300-400	10 (5.0)		14 (4.4)			
	400-500	10 (5.0)		16 (5.0)			
	>500	10 (5.0)		28 (8.8)			
Clinics without beds, n (%)			806 (80.0)		1073 (77.1)		.09
**Location, n (%)**							
	In the prefecture	552 (54.8)		626 (45.0)		<.001	
	Out of the prefecture	455 (45.2)		766 (55.0)			

^a^Not applicable.

^b^The Japanese Medical Care Act defines medical institutions with over 20 beds as hospitals and those with fewer than 20 beds as clinics.

**Table 4:** Results of the interrupted time-series analysis (ITSA) for the immediate change and changes in the trend.

Subsample	RR^a^ (immediate change at reference time point)	95% CI	*P* value	Change in trend	95% CI	*P* value
Telehealth users overall	2.97	2.14-2.31	<.001	1.00	1.00-1.01	<.001
≥65 years old	2.98	2.89-3.07	<.001	1.01	1.00-1.00	<.001
<65 years old	2.93	2.76-3.12	<.001	1.03	1.02-1.03	<.001

^a^RR: rate ratio.

**Table 5:** Comparison of model estimates with the sensitivity analysis.

Sensitivity case	RR^a^	95% CI	Change in trend	95% CI	AIC^b^
No removal	2.27	2.22-2.32	0.99	0.99-0.99	8390.9
Removal bandwidth = 1 (base model)	2.97	2.14-3.31	1.00	1.00-1.01	1122.3
Removal bandwidth = 2	2.94	2.86-3.02	1.01	1.01-1.02	1106.4
Removal bandwidth = 3	2.98	2.88-3.09	1.01	1.01-1.01	968.5

^a^RR: rate ratio.

^b^AIC: Akaike information criterion.

## Discussion

### Principal Findings

Table 1 presents the basic information about patients who used telehealth. During the observation period, 13,618 patients (33.71 per 1000 beneficiaries, 18.62 per 1000 outpatients) used telehealth before April 2020, and 28,853 (71.42 per 1000 beneficiaries, 39.46 per 1000 outpatients) did so after that time. An immediate increase after the reference time point was also significantly evident with ITSA (RR 2.97; 95% CI 2.14-2.31). However, no change in the trend was observed (change in trend 1.00; 95% CI 1.00-1.01). Among the patients who used telehealth, there was a higher proportion of women than men. After the reference point, there were increases in the frequency of telehealth use per patient, the number of telehealth users for initial consultations, and medical expenses. Regarding diseases and conditions associated with telehealth use, we observed an increase in hyperlipidemia, cardiovascular disease, hyperuricemia, and mental disease. Most telehealth services were provided by clinics, but we identified an increase in the number of hospitals using telehealth. We observed an increase in the proportion of telehealth utilization in medical institutions outside Mie Prefecture compared with inside the prefecture.

Recent studies have reported an increase in telehealth use with COVID-19 [17,23]. However, no reports have examined how telehealth utilization has changed in a country such as Japan, where patients have free access to medical care and facilities are abundant but where the government has imposed restrictions on telehealth. With ITSA, this study confirmed the immediate increase in telehealth use after April 2020. However, no following change in the trend was evident with either model. This is not consistent with results from a previous study in the United Kingdom, where universal health coverage is provided through the National Health Service, similar to Japan [24]. When focusing on the Japanese policy strategy for telehealth from the point of view of providers, until at least 2022, telehealth has not spread widely in Japan because face-to-face consultations are more profitable for medical institutions than telehealth. Therefore, to expand the use of telehealth, not only deregulation but also a review of the incentive system are considered necessary. In addition, since deregulation of telehealth was a temporary measure until April 2022, medical institutions’ use of telemedicine may have been inhibited. This should also be researched, by extending the observation period.

We should state that our database did not include all patients; employee-insured patients, for example, were excluded. Our database mainly covered the population who were older than 65 years, which allowed us to capture the impact of telehealth utilization on this group. Nevertheless, we obtained consistent results when the ITSA was conducted with a subgroup analysis—with patients divided into those aged under 65 years and those aged 65 years and older. Whether similar results can be obtained for the entire population younger than 65 years of age is a matter for additional analysis.

We found that telehealth use was mostly by telephone and for prescriptions. Such utilization has the potential to serve as an alternative to conventional consultations, but additional verification of patient safety is required. We observed that online consultation was rarely used by public insurance services. In the Japanese health care system, telehealth is mainly limited to follow-up purposes, and it is likely that telephone consultation was chosen because many cases had little need for face-to-face consultation. Since online consultation is a relatively new system in Japan, having been issued in 2018, it is necessary to continue to investigate its prevalence. In addition, the Japanese government’s deregulation of telehealth in April 2020, which was announced as a temporary measure, may have slowed the increase in the number of medical institutions intending to adopt telehealth or caused them to defer decisions about it.

With respect to telehealth use, we observed changes in patient characteristics and in disease composition as well as increased medical costs after the time reference point. Our results suggest that the Japanese government should not only examine the spread of telehealth in terms of the increased number of cases but also focus on changes in the characteristics of patients who use telehealth. If drug treatment becomes the main treatment strategy, medical resource allocation should be considered in conjunction with pharmacy allocation. The complementary role of telehealth should also be evaluated: It is difficult to provide only telehealth when physical treatment, such as nutritional guidance and rehabilitation, is involved.

Japanese patients are not generally treated by primary care physicians or general practitioners; not all patients have specific physicians they consult on a regular basis. Given that more patients are using telehealth for initial consultations, it is necessary to promote a communication infrastructure that offers the following: vetting of eligible patients, clarification of criteria for the type of treatment (changing to face-to-face consultations), and collection of the required information. With regard to the type of medical institutions providing telehealth, the number of hospitals has increased; thus, the government needs to design a system that takes into account changes in providers. The increasing use of medical institutions outside the patient’s prefecture of residence suggests that access to telehealth is affected by factors other than geography.

### Limitations

One strength of the present study was identifying the use of telehealth and how it changed after COVID-19 in part of Japan, which has free access to medical care and abundant medical facilities. However, several limitations of this study deserve mention. First, this was an observational study limited to Mie Prefecture. Therefore, caution should be exercised regarding the applicability of our results to the general population; our results should be verified for Japan as a whole. Second, this study did not cover all insured individuals, which may have led to selection bias. In particular, it should be noted that our results strongly reflected the trends among older adults; there was a lack of information on telehealth use among people enrolled in employee insurance (ie, the population of working age). Third, several studies have reported better outcomes in improving treatment adherence and preventing serious events with telehealth use [25-27]. Longer observation periods than in the present study would be needed to determine the incidence of events that could serve as medical outcomes; thus, this is a matter for future study. Finally, we observed changes in patient and health care provider characteristics and trends only as a temporary state. However, it is possible that circumstances after COVID-19 will continue to change dynamically. Therefore, further data collection and analysis over a longer period are needed.

### Conclusions

The observations of this study from January 2017 to September 2021 indicate that telehealth use temporarily increased after COVID-19; however, the diffusion trend remained unchanged. The attributes of patients who used telehealth and the medical institutions that provided it underwent change: Decision makers for related policies need to design their systems in light of this environment.

## Appendix

Multimedia Appendix 1 Supplemental material.

## Abbreviations

AIC: Akaike information criterion
ICD-10: International Classification of Diseases 10th Revision
ITSA: interrupted time-series analysis
RR: rate ratio

## Data Availability

The data set can be utilized for limited use, and its sharing with third parties is not allowed but, if needed, will be shared on request with the permission of the regional insurers in Mie, Japan.
