# Supplementary material for: The Association Between Telehealth Utilization and Policy Responses on COVID-19 in Japan: Interrupted Time-Series Analysis
Source: Interact J Med Res. 2022 Jul 12;11(2):e39181. doi: 10.2196/39181 (PMC9278403; doi:10.2196/39181)
Supplement: Multimedia Appendix 1 [file ijmr_v11i2e39181_app1.docx]

SUPPLEMENTAL MATERIAL

**Figure S1. Autocorrelation and partial autocorrelation plots of the Ratio of Telemedicine Users to Outpatients**


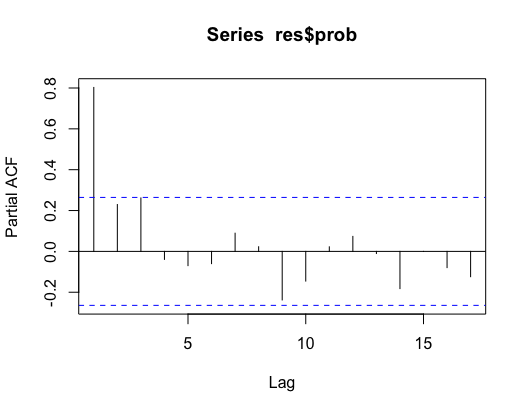

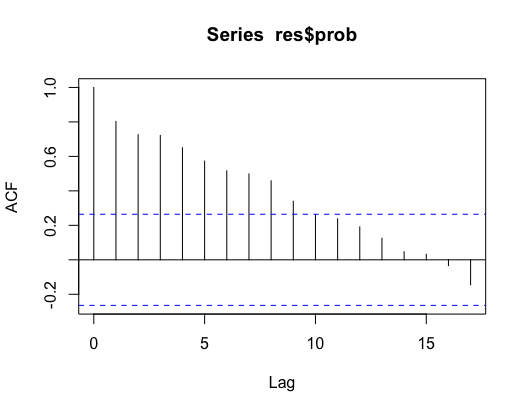


A

B

Figure S1 shows autocorrelation plot (A) and partial autocorrelation plot (B) of the observed data. The blue dotted lines indicate the upper/lower limits of the 95% confidence interval.

**Figure S2. Autocorrelation and partial autocorrelation plots of residuals between predicted and observed values**


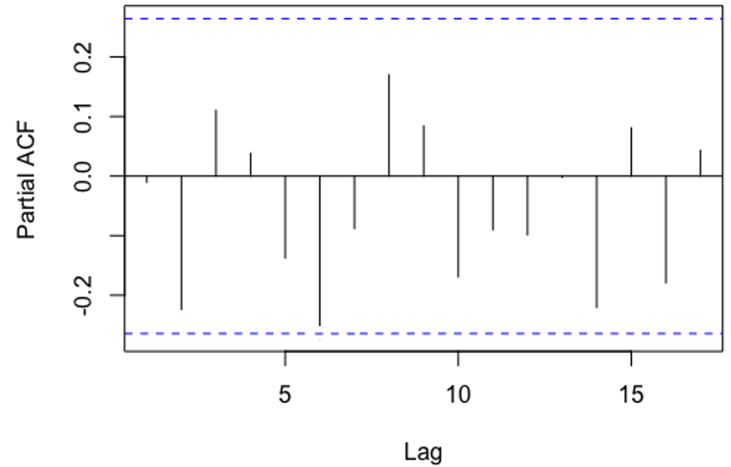

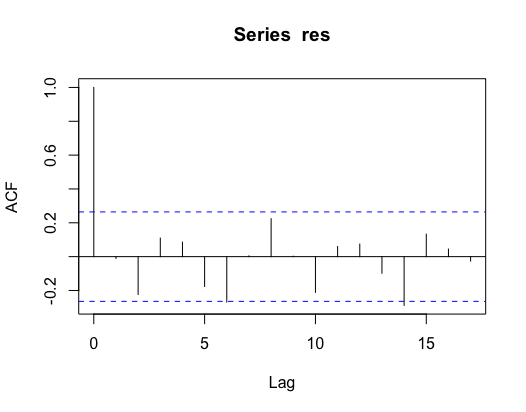


A

B

Figure S2 shows autocorrelation plot (A) and partial autocorrelation plot (B) of the residuals between predicted and observed values are shown . The blue dotted lines indicate the upper/lower limits of the 95% confidence interval.

**Table S1. COVID-19 confirmations and deaths, monthly data from receipt database in the Mie Prefecture.**

| **Year** | **Month** | **Confirmed case with COVID-19[1]** | **Deaths related to COVID-19[1]** | **Telehealth Users** | **Outpatients** | **Beneficiaries** |
| --- | --- | --- | --- | --- | --- | --- |
| 2020 | January | 1 | 0 | 1,079 | 416,578 | 728,754 |
| 2020 | February | 0 | 0 | 1,122 | 413,306 | 729,647 |
| 2020 | March | 10 | 0 | 2,064 | 415,141 | 728,331 |
| 2020 | April | 34 | 1 | 5,724 | 403,955 | 731,226 |
| 2020 | May | 0 | 0 | 5,083 | 386,371 | 734,071 |
| 2020 | June | 1 | 0 | 3,173 | 411,326 | 734,376 |
| 2020 | July | 55 | 0 | 3,164 | 414,849 | 735,067 |
| 2020 | August | 279 | 1 | 3,841 | 404,035 | 736,359 |
| 2020 | September | 129 | 5 | 3,459 | 407,895 | 740,827 |
| 2020 | October | 56 | 0 | 2,960 | 421,042 | 746,374 |
| 2020 | November | 293 | 0 | 2,952 | 407,470 | 750,976 |
| 2020 | December | 433 | 11 | 3,877 | 416,096 | 754,833 |
| 2021 | January | 897 | 12 | 4,237 | 396,650 | 759,085 |
| 2021 | February | 340 | 23 | 3,555 | 399,756 | 763,083 |
| 2021 | March | 225 | 17 | 3,730 | 419,974 | 765,858 |
| 2021 | April | 1,012 | 11 | 4,066 | 417,753 | 774,852 |
| 2021 | May | 1,148 | 25 | 3,679 | 405,174 | 778,573 |
| 2021 | June | 343 | 6 | 3,712 | 415,918 | 781,013 |
| 2021 | July | 506 | 2 | 3,354 | 415,218 | 784,020 |
| 2021 | August | 6,618 | 16 | 4,027 | 409,105 | 787,375 |
| 2021 | September | 2,237 | 32 | 3,928 | 407,579 | 791,137 |

Table S1 shows information of COVID-19 prevalence in the study areas, the Mie prefecture, Japan, to grasp association between telehealth use and prevalence. This shows correlation between the change of confirmed cases number and telehealth use does not identify (The correlation coefficient between both variables is not significant).

Referces

1. Mie Prefecture. Medical and Public Health Department. Outbreak of new coronavirus infection (Japanese Only). URL: https://www.pref.mie.lg.jp/YAKUMUS/HP/m0068000066_00071.html. [Accessed May 6, 2022].
